# Genome-wide Analysis Reveals DNA Methylation Alterations in Obesity Associated with High Risk of Colorectal Cancer

**DOI:** 10.1038/s41598-019-41616-0

**Published:** 2019-03-25

**Authors:** Lixin Dong, Li Ma, Gloria H. Ma, Hongmei Ren

**Affiliations:** 1 Mumetel LLC, University Technology Park at IIT, Chicago, IL USA; 2 Molecular Pathology, ACL Laboratories, Advocate Health Care, Rosemont, IL USA; 3 Department of Pathology, Advocate Sherman Hospital, Elgin, IL USA; 4 Department of Biochemistry & Molecular Biology, Wright State University, 3640 Colonel Glenn Hwy., Dayton, OH USA

## Abstract

Obesity is a high risk factor for colorectal cancer (CRC). The contribution of underlying epigenetic mechanisms to CRC and the precise targets of epigenetic alterations during cancer development are largely unknown. Several types of epigenetic processes have been described, including DNA methylation, histone modification, and microRNA expression. To investigate the relationship between obesity and CRC, we studied both obese and CRC patients, focusing on genome-wide peripheral blood DNA methylation alterations. Our results show abnormal distributions of overlapping differentially methylated regions (DMRs) such as hypermethylated CpG islands, which may account for epigenetic instability driving cancer initiation in obesity patients. Furthermore, functional analysis suggests that altered DNA methylation of extracellular (e.g., O-glycan processing) and intracellular components contribute to activation of oncogenes (e.g. KRAS and SCL2A1) and suppression of tumor suppressors (e.g. ARHGEF4, EPHB2 and SOCS3), leading to increased oncogenic potency. Our study demonstrates how DNA methylation changes in obesity contribute to CRC development, providing direct evidence of an association between obesity and CRC. It also reveals the diagnostic potential of using DNA methylation as an early risk evaluation to detect patients with high risk for CRC.

## Introduction

Being overweight or obese is considered to be a major risk factor for many cancers, in particular colorectal cancer (CRC)^1–3^. Epidemiological data suggests that obesity is associated with a 1.2–2.0 fold increased risk of CRC^4^. Even though the close link between obesity and the risk of CRC has been suggested by a large number of studies^5–9^, the underlying molecular mechanisms are still largely unknown. Understanding the mechanisms linking obesity to the development of CRC may lead to the development of accurate methods for early detection and the identification of new targets for CRC prevention.

DNA methylation is an epigenetic mechanism that occurs when a methyl group is added onto the C5 position of cytosine, thereby modifying gene function and affecting gene expression^10–12^. Most DNA methylation occurs at cytosine residues that precede guanine residues, called CpG dinucleotides, which tend to cluster in DNA domains known as CpG islands. The relationship between methylation and gene expression is complex. In general, DNA methylation of gene promoters is associated with transcriptional silencing^13^, whereas methylation in gene bodies is associated with increased gene expression^14–16^. Strong correlations between gene expression and CpG islands and island shores have been demonstrated^17^. Inappropriate methylation of CpG islands could result in impaired transcription factor binding, recruiting repressive methyl-binding proteins, and stably silencing gene expression^10^. Global hypomethylation is thought to influence CRC development by inducing chromosomal instability^18–20^.

Compared to studies in cancer, studies in obesity have not provided consistent evidence of a role for global methylation changes. Furthermore, differentiating early epigenetic alterations potentially involved in cancer initiation is difficult considering the influence of multiple other factors on these epigenetic changes. Consequently, studying specific methylation changes that affect oncogenic transformation signaling is likely to provide a better picture of the association between obesity and CRC development.

Genome-wide mapping of differentially methylated CpG sites (DMCs) or differentially methylated regions (DMRs) is an important means to reveal the impact of epigenetic modifications on inheritable phenotypic variation in both obesity and CRC and to understand their correlation. Currently, a massive effort is directed at providing better insight into tissue-specific epigenetic alternations and their roles in disease development^21–25^. Ronn, T. *et al*. demonstrated that epigenetic biomarkers in blood can mirror epigenetic signatures in target tissues^21^. Using bisulfite pyrosequencing, Ally and colleagues observed a correlation between colonic tissue methylation and blood methylation of estrogen receptor 1 (ESR1) that is independent of age, gender, disease status, and body mass index (BMI)^26^. To date, only a few studies have reported results from examining the genome-wide methylation pattern in colorectal tumors^27–30^ and no earlier studies have specifically addressed the effects of DNA methylation alterations in the blood of CRC patients. The aim of the present study was to explore whole blood DNA methylation patterns in obese and CRC patients to identify epigenetic changes associating CRC to obesity by comparing whole genomic DMR and DMC patterns of DNA methylation using an overlapping method. We provide direct evidence of the connection between cancer development and obesity. The recognition that the same epigenetic changes are a driving force for the development into CRC in obese individuals supports the promising biomarker potential of DNA methylation studies for early diagnosis.

## Results

### Significant associations observed between obesity and CRC in Overlapping DMCs and DMRs

**Figure 1:**
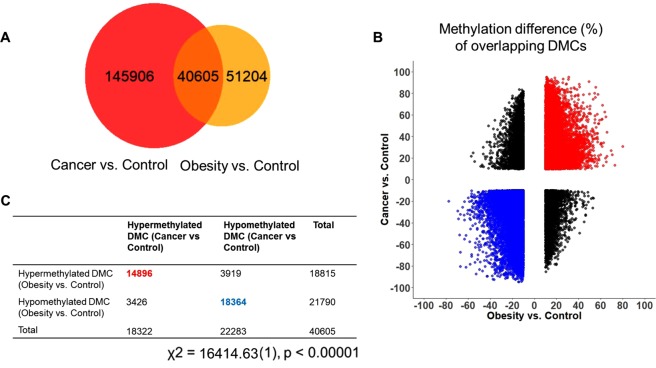
Association of differentially methylated CpGs (DMCs) in obesity and CRC. (**A**) Venn-diagram of DMCs generated from CRC vs. Control and Obesity vs. Control genes; (**B**) Scatter plot displaying the methylation differences in overlapping DMCs and the distribution of these DMCs partitioned by hyper-/hypomethylated CpGs in CRC and obesity; (**C**) Number of hyper-/hypomethylated CpGs in CRC and obesity. The Chi-Square test was used to determine a potential significant relationship between obesity and CRC.

Genome-wide methylation analysis was conducted in 15 CRC patients and compared to publically available data from 10 obese subjects and 15 healthy lean controls (Table 1). The case and control groups were comparable with respect to gender. Age was used as a covariate in differential analysis in order to remove its possible effects. To avoid systematic errors for the DNA methylation data, histogram transformation was applied to equalize the distributions of the methylation levels to the control group. We performed differential methylation analysis of the reduced representation bisulfite sequencing (RRBS) profiling data for the obesity and CRC cases versus the control group. We identified 186,511 DMCs between CRC and control subjects and 91,809 DMCs between obese and controls (Fig. 1). To evaluate whether obesity is associated with CRC through DNA methylation alterations, we overlapped these DMCs identified separately from CRC and obesity. If CRC and obese DNAs were both differentially methylated at a certain CpG site, the DMC was counted as an overlapping DMC. As shown in Fig. 1A, there were 40,605 overlapping DMCs, accounting for 44% of DMCs identified from the obese group. Surprisingly, we observed methylation changes of these overlapping DMCs occurred in the same direction for obesity and CRC (Fig. 1B**)**. In other words, a vast majority of overlapping DMCs for obesity and CRC were either hypermethylated (36.7%) or hypomethylated (45.2%) for both obesity and CRC (Fig. 1C**)**. A Chi-square test showed that there was a significant association between obesity and CRC (p < 0.0001).

**Table 1:** Subject Characteristics.

		Control n = 15	Obesity n = 10	CRC n = 15
Age	(Mean ± SD)	40 ± 15 years	36 ± 10 years	53 ± 9 years
	Range	(21–65)	(23–52)	(39–71)
Gender	F	7	5	8
	M	8	5	7
BMI	(Mean ± SD)	22.9 ± 3.5 kg/m^2^	34.4 ± 4.1 kg/m^2^	28.1 ± 5.6 kg/m^2^

**Figure 2:**
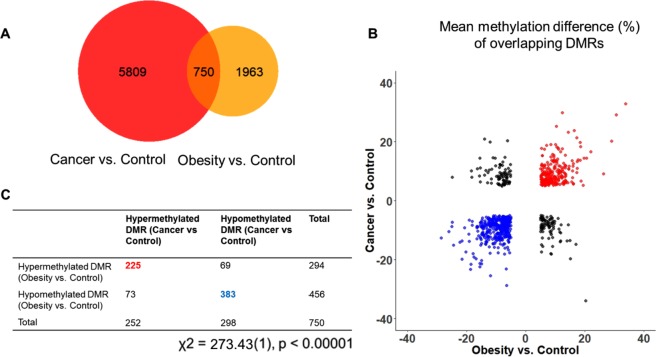
Association of differentially methylated regions (DMRs) in obesity and CRC. (**A**) Venn-diagram of the DMRs generated from CRC vs. Control and Obesity vs. Control genes; (**B**) Scatter plot displaying the mean methylation difference of overlapping DMRs and the distribution of these DMRs partitioned by hyper-/hypomethylated DMRs in CRC and obesity. (**C**) Number of hyper-/hypomethylated DMRs in CRC and obesity.

A similar trend of DNA methylation alterations was observed in the 750 overlapping DMRs identified (Fig. 2). They accounted for 27.6% of the 2,713 DMRs identified in the obese group. The association between obese and CRC in overlapping DMRs was highly significant as suggested by Chi square test (p < 0.0001). These data suggest that obesity is highly associated with the risk of developing CRC.

### Distribution patterns in overlapping DMRs is Similar to the pattern in CRC

**Figure 3:**
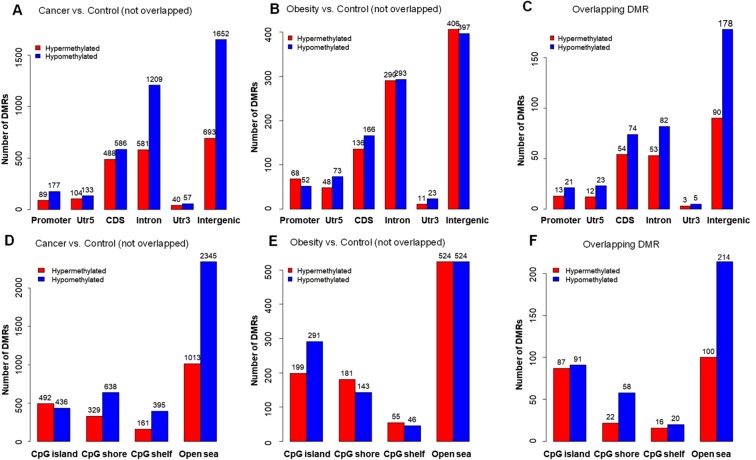
Bar plots of the number of cancer-specific, obesity-specific, overlapping DMRs by gene subregions (**A–C**) and CpG islands (**D–F**).

We then annotated hypermethylated or hypomethylated overlapping DMRs for both obesity and CRC to gene regions and CpG islands (Fig. 3). In CRC, peripheral blood showed higher overall genomic hypomethylation than hypermethylation^21–24^ (Fig. 3A). This is consistent with previous reports showing that genomic DNA hypomethylation is a hallmark of most cancer genomes, prompting genomic instability and cancer transformation^20,25–28^. In contrast, the numbers of hyper- or hypomethylated DMRs in all gene regions were similar in obesity (Fig. 3B). However, the distribution of overlapping DMRs (Fig. 3C) was closer to the distribution of non-overlapping DMRs in cancer than the distribution of non-overlapping DMRs in obesity. Greater number of hypomethylated rather than hypermethylated DMRs were identified in the promoter, intron and intergenic regions in cancer and overlapping DMRs (Fig. 3A,C). Similarly, a closer distribution was seen among the distribution of overlapping DMRs and the distribution of non-overlapping DMRs in cancer over CpG islands (Fig. 3D–F).

These data suggest that the risk of obesity-related CRC can be potentially be evaluated by analyzing overlapping DMRs. Using gene annotation enrichment analysis, biomarkers linking obesity to CRC risk can be potentially identified.

### Functional analysis of genes associated with overlapping DMRs

To identify the most important DMRs linking obesity to CRC, DMRs either hyper- or hypomethylated for both obesity and CRC were further selected using nonparametric procedures. The average methylation level across DMRs was calculated for each subject, and the Kruskal-Wallis test, followed by multiple pair-wise comparisons of groups by Mann-Whitney U test, was performed to determine significance of differences. Four hundred and forty-two DMRs with a p value of at least 0.1 or less by the Kruskal-Wallis and Mann-Whitney U tests were selected. Among these, 238 DMRs were located in the promoter or gene body regions.

KEGG pathway^29–31^ and GO biological process (GO-BP) analyses of the 238 genes associated with overlapping DMRs were performed to better understand how DNA methylation links obesity to CRC development. The top KEGG pathways and GO-BP are summarized in Table 2. Overall, these analyses, enriched by selected genes, are related to the extracellular microenvironment such as the extracellular matrix microbiota and mucin glycans, and to changes in intracellular signaling pathways, such as metabolic, transforming growth factor (TGF)-β and KRAS signaling, which may play a central role in CRC initiation^32,33^.

**Table 2:** KEGG pathways and Gene Ontology-Biological Processes (GO-BP) enriched for genes with DMRs.

GO/KEGG ID	Biological process/KEGG pathway	Gene count	P-value	Genes
GO0001525	Angiogenesis	10	0.001	PRKD2, EGFL7, FAP, BCAS3, PLXND1, MYH9, ADAM8, TMPRSS6, TGFB2, EPHB2
GO0071230	Cellular response to amino acid stimulus	5	0.002	EGFR, SH3BP4, COL6A1, RPTOR, NEURL1
GO0007411	Axon guidance	7	0.012	KRAS, WNT3, EFNA2, KIF26A, CDH4, TGFB2, EPHB2
GO0006508	Proteolysis	13	0.017	CAPN15, DHH, CAPN10, THOP1, FAP, CAPN9, RHBDF1, ST14, DPEP3, HTRA3, ADAM8, PMPCA, TMPRSS6
GO0043547	Positive regulation of GTPase activity	14	0.018	ARHGEF4, EGFR, OBSCN, LIMS1, ARHGEF7, CAMK2G, RASAL1, ACAP3, TBCD, BCAS3, RAP1GAP2, SHC2, EPS8L1, FGF3
GO0043393	Regulation of protein binding	3	0.021	HDAC4, SMARCD3, PAX7
GO0021772	Olfactory bulb development	3	0.021	CRTAC1, EFNA2, SKI
GO0007399	Nervous system development	9	0.022	MYT1L, HDAC4, ARHGEF7, CAMK2G, PCDHB12, IGSF9B, DPF1, NEURL1, EPHB2
GO0030198	Extracellular matrix organization	7	0.030	COL9A1, COL9A3, ITGAX, ICAM5, ELN, COL6A1, TMPRSS6
GO0016266	O-glycan processing	4	0.035	GALNT6, GCNT1, MUC5B, B4GALT5
GO0051491	Positive regulation of filopodium assembly	3	0.038	PALM, BCAS3, NEURL1
GO0045930	Negative regulation of mitotic cell cycle	3	0.038	EGFR, BRINP1, SMAD3
GO0007156	Homophilic cell adhesion via plasma membrane adhesion molecules	6	0.041	CDH12, SDK1, PCDHB12, IGSF9B, CDH4, KIRREL3
GO0030512	Negative regulation of transforming growth factor beta receptor signaling pathway	4	0.041	SMAD3, SKI, HTRA3, LDLRAD4
GO0002520	Immune system development	2	0.047	SMAD3, CACNA1C
GO0032909	Regulation of transforming growth factor beta2 production	2	0.047	SMAD3, TGFB2
GO0001938	Positive regulation of endothelial cell proliferation	4	0.049	PRKD2, EGFL7, NR4A1, RPTOR
hsa00512	Mucin type O-Glycan biosynthesis	4	0.008	WBSCR17, GALNT6, GCNT1, B4GALT5
hsa04550	Signaling pathways regulating pluripotency of stem cells	7	0.011	SETDB1, KRAS, WNT3, PCGF3, APC2, SMAD3, WNT2B
hsa04925	Aldosterone synthesis and secretion	5	0.023	PRKD2, CAMK2G, NR4A2, NR4A1, CACNA1C
hsa04020	Calcium signaling pathway	7	0.032	EGFR, GNAL, CAMK2G, RYR3, NTSR1, CACNA1C, PTAFR
hsa05206	MicroRNAs in cancer	9	0.037	EGFR, KRAS, WNT3, APC2, ST14, MIR133A2, ABCC1, RPTOR, TGFB2
hsa05210	Colorectal cancer	4	0.050	KRAS, APC2, SMAD3, TGFB2
hsa04921	Oxytocin signaling pathway	6	0.050	EGFR, KRAS, CAMK2G, RYR3, CACNA1C, CACNA2D4

KEGG, Kyoto Encyclopedia of Genes and Genomes; GO, gene ontology.

### Major pathways affected by DNA methylation

**Figure 4:**
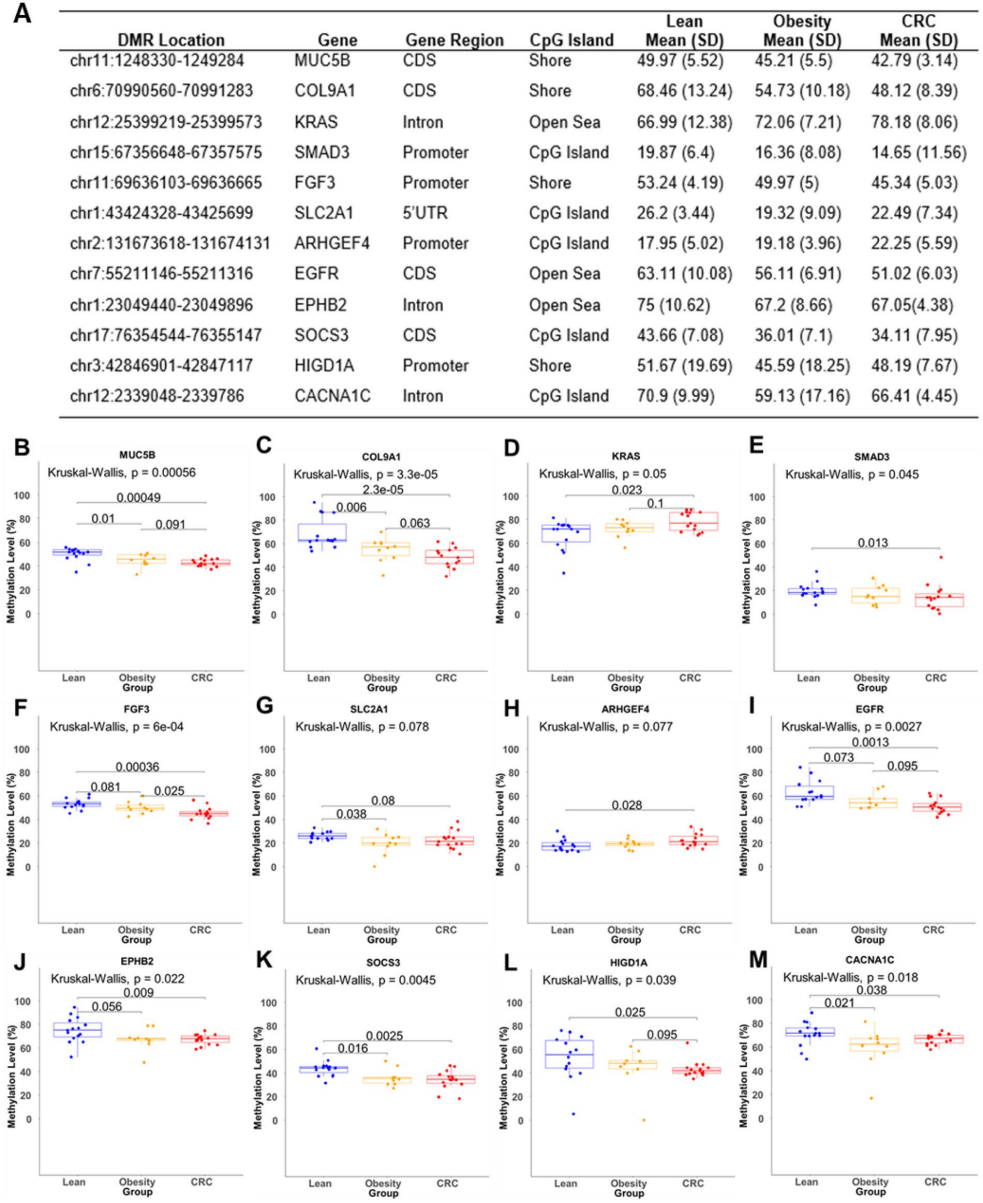
Mean methylation levels of relevant DMRs. (**A**) Summary of 12 overlapping major DMRs; (**B–M**) Box plots of methylation level of individual DMRs in three different gene groups. Each dot represents the mean methylation level of the specific DMR for each individual subject.

The signal-transduction pathways dysregulated by DNA methylation changes in both obesity and CRC include: 1) extracellular matrix components, i.e., O-glycan processing, protein glycosylation, and extracellular matrix scaffold; 2) KRAS and TGF-β signaling; and 3) lipid and glucose metabolism (Table 2 and Fig. 4). The alterations in these extracellular and intracellular metabolites could induce CRC-associated metabolic reprogramming in obesity and contribute to the initiation of CRC in obese patients. Further details are given below.

#### Extracellular matrix components affected by methylation changes

In the O-glycan processing (GO: 0016266) and mucin type O-glycan biosynthesis pathway (hsa00512), 5 genes were associated with selected DMRs. β-1,4-galactosyltransferase 5 (B4GALT5) (intron region), polypeptide N-Acetyl galactosaminyl transferase 17 (GALNT17) (intron region) and mucin 5B (MUC5B) (coding region) (Fig. 4B). These genes were hypomethylated, whereas glucosaminyl (N-acetyl) transferase 1 (GCNT1), GALNT6 were hypermethylated in the 5′-UTR and promoter regions, respectively. The CRC microenvironment and extracellular matrix are mainly constituted by collagen and elastin^34–36^. In the extracellular matrix organization (GO: 0030198), we observed that the elastin (ELN), and collagen α1 (IX) chain (COL9A1) (Fig. 4C) in the coding region were hypomethylated, and COL6A1 was hypermethylated in the coding region. Elastin and collagen are the main components of elastic fibers and their DNA methylation changes indicate altered extracellular matrix barrier function which may be associated with tumor progression^37,38^.

#### Altered DNA methylation of KRAS and TGF-β signaling

In the pathways in cancer (hsa05200), although not in the list of top KEGG pathways, we found 10 genes with selected DMRs, among which 4 genes are involved in the CRC pathway (hsa05210) (see Table 2). These include TGF-β2, KRAS (Fig. 4D), adenomatous polyposis coli protein 2 (APC2) and SMAD family member 3 (SMAD3) (Fig. 4E). KRAS, a well-established proto-oncogene, was hypermethylated in the intron region. We also found that fibroblast growth factor 3 (FGF3) (Fig. 4F), upstream of KRAS, is associated with a hypomethylated DMR in its promoter region in obesity and is even further hypomethylated in CRC. SMAD3, a central component of the TGF-β signaling pathway^39^, was hypomethylated in the promoter region. Solute carrier family 2 member 1 (SLC2A1), which encodes the glucose transporter type 1 protein (GLUT1) and is responsible for basal glucose transport in all cell types, contained a hypomethylated DMR in its 5′-UTR region **(**Fig. 4G**)**. APC-stimulated guanine nucleotide-exchange factor (ARHGEF4) was hypermethylated in the promoter region in CRC peripheral blood **(**Fig. 4H**)**. The epidermal growth factor receptor (EGFR), a transmembrane tyrosine kinase involved in triggering the MAPK signaling pathway^40^, was associated with a hypomethylated DMR in its coding region (Fig. 4I). Eph receptor B2 (EPHB2) has been suggested to be a tumor suppressor gene in colorectal carcinogenesis^41^, and was associated with a hypomethylated DMR in its intron region (Fig. 4J).

#### Aberrant DNA methylation of lipid and glucose metabolism genes

Obesity is related to energy imbalance and metabolic dysfunction^42,43^. Consistent with this, we observed some related genes associated with overlapping DMRs. Regulatory-associated protein of mTOR (RPTOR) is involved in the control of mTORC1 activity, which plays an important role in lipogenesis and in regulating the endothelial cell proliferation (GO: 0001938) (see Table 2). We identified hypermethylated overlapping DMR of RPTOR located in the gene body region. In addition, we observed a hypomethylated DMR in the coding region of suppressor of cytokine signaling 3 (SOCS3) (Fig. 4K). We also observed methylation changes in several mitochondria-related genes, such as hydroxyacyl-Coenzyme A dehydrogenase (HADH), an enzyme that catalyzes the metabolism of short-and medium-chain fatty acids^44^. This showed a hypomethylated DMR in the gene body region in obesity which was further hypomethylated in CRC, representing changes that may affect its function in lipid metabolism. The succinate dehydrogenase (SDH) complex (SDHAF1), which encodes a protein essential for the assembly of mitochondrial enzyme succinate dehydrogenase (SDH), the main element of complex II^45^, was hypermethylated in its promoter region in obesity and further hypermethylated in CRC. In addition, RXRA, a common heterodimeric partner for a number of nuclear receptors^46^, was hypermethylated in the intron region in obesity and further hypermethylated in CRC. Consistent with a recent study showing that HIGD1A expression is increased during glucose deprivation to modulate cell survival and tumor growth^47^, we observed a hypomethylated DMR in the promoter region of HIGD1A in CRC (Fig. 4L). Calcium voltage-gated channel subunit alpha1 C (CACNA1C) belongs to the insulin secretion and MAPK signaling pathways and alterations in its expression may have an adverse effect on tissue homeostasis, which may result in tumorigenesis^48^. We identified a hypomethylated DMR in the intron region (Fig. 4M). Collectively, aberrant lipogenesis and changes in lipid and glucose metabolism are key features of metabolic reprogramming, which may induce aberrant activation of KRAS signaling and a sustained pro-inflammatory environment, leading to cancer initiation.

#### Altered mRNA expression of representative genes affected by DNA methylation in HCT116 cells

**Figure 5:**
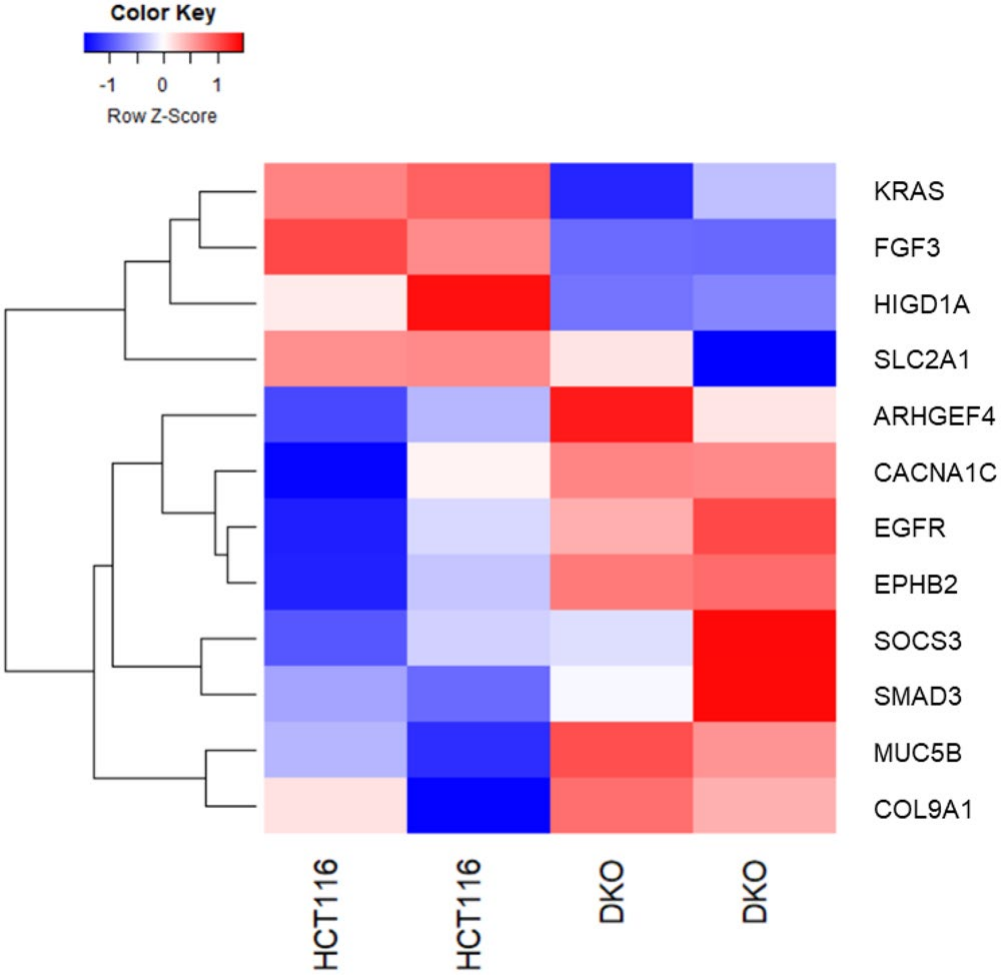
Heatmap displays mean row-centered log-CPM (log2-counts per million) values of the representative genes selected in Fig. 4 in two replicas of HCT116 and DKO cell line.

The DNA methyltransferases, DNMT1 and DNMT3B, are essential for maintenance and de novo CpG methylation and disruption of these 2 genes results in more than 95% loss of genomic methylation^49^. To further confirm the functional impact of DNA methylation alterations on gene expression, we utilized two human colorectal carcinoma cell lines, namely HCT116 wild type and HCT116 DNMT1/DNMT3B double knockout (DKO) cells. As shown in Fig. 5, gene expression of these representative genes were affected by DNA methylation changes with high reproducibility. We observed an opposite effect on the gene expression of a group of genes (KRAS, FGF3, HIGD1A and SLC2A1) as compared with the other group of genes (ARHGEF4, CACNA1C, EGFR, EPHB2, SOCS3, SMAD3, MUC5B and COL9A1). Especially, KRAS showed reduced expression and ARHGEF4 showed elevated expression in HCT116 DKO as a consequence of DNMT inhibition, consistent with the hypermethylated DMRs we observed in the gene body and promoter regions of KRAS and ARHGEF4, respectively in CRC.

## Discussion

CRC remains the second leading cause of cancer-related death in the United States^50^. The 5-year relative survival rate for early-stage CRC is 90%; for advanced stage IV CRC, the rate drops to about 11%^51^. But only about 4 out of 10 CRCs are found at the early stage^52^, partially due to the poor patient acceptance and/or sensitivity of available screening modalities. Blood-based DNA methylation has great potential as an early, accurate, non-invasive biomarker for risk evaluation and early detection to improve the survival rate for CRC patients. Obesity is a complex disorder that contributes to many human diseases^53^. In this study, we aimed at understanding whether DNA methylation alterations in blood play a role in the association between obesity and CRC. Using genome-wide methylation sequencing data and overlapping analysis, we observed DNA methylation changes in obesity and CRC with significant association (Figs 1 and 2). The distribution pattern of overlapping DMRs, such as hypermethylated CpG islands, was similar as the pattern of DMRs in CRC were comparable to that in obesity (Fig. 3). By further analyzing these overlapping DMRs, we observed DNA methylation changes in extracellular matrix components and organization, O-glycan processing, and intracellular factors including KRAS signaling and lipid and glucose metabolism, all pathways that may enhance the CRC risk in obesity.

In the extracellular components, we showed DNA methylation changes in the mucin type O-glycan biosynthesis pathway (hsa00512) and O-glycan processing (GO: 0016266) (Table 2). Mucins are the main components of mucus and the colonic mucus forms a protective homeostatic barrier against enteric pathogens between the resident microbiota and the underlying epithelial cells^54–57^. These DNA methylation alterations would lead to mucus degradation and compromise epithelial barrier function.

In the intracellular signaling domain, our data indicated altered DNA methylation of KRAS and of metabolic reprogramming, which play a crucial role in tumorigenesis. Our data suggests that metabolic stress in obesity contributes to the acquisition of an oncogenic potential. Alterations in DNA methylation may contribute to dysregulation of the insulin signaling pathway, which is associated with activated oncogenes (e.g. KRAS and SCL2A1) and downregulated tumor suppressors (e.g. SOCS3, EPHB2 and ARHGEF4), leading to increased and unregulated cellular proliferation and malignant transformation. The intron region of KRAS was hypermethylated in both CRC and obesity. KRAS signaling is also a shared component in signaling pathways regulating pluripotency of stem cells, microRNAs in cancer and oxytocin signaling pathway (hsa04550, hsa05206, and hsa04921, respectively), suggesting its central role in obesity and cancer pathology. SCL2A1, which encodes the GLUT1 transporter^58^, was hypomethylated in its promoter region. GLUT1 is primarily undetectable in normal epithelial tissues and benign epithelial tumors, and overexpression of GLUT1 during oncogenesis has been identified in various cancers, and is considered as an important player of active tumor cell glucose uptake and metabolism^59,60^. In addition, metabolic stress, such as increased glucose uptake induced by SLC2A1 upregulation and glycolysis, is consistent with oncogenic mutations in oncogenes, such as KRAS or BRAF^61^. In contrast, the coding region of SOCS3 which is associated with obesity-related cancers, was hypomethylated in obesity and CRC. A previous study suggested that methylation silencing of SOCS3 suppresses its response to IL-6 stimulation and increases the propensity to malignant transformation^62^. ARHGEF4 is a binding partner of adenomatous polyposis coli (APC)^63^ and an important tumor suppressor gene in the development of CRC^64^. We found that ARHGEF4 was hypermethylated in the promoter region, which may suppress its expression. Moreover, we observed the transforming growth factor (TGF)-β /SMAD signaling was disrupted by DNA methylation as a shared component in angiogenesis, negative regulation of TGF-β receptor signaling pathway, regulation of TGF-β production and CRC pathway (GO: 0001525, GO: 0030512, GO: 0032909 and hsa05210, respectively). This disruption may contribute to pro-tumorigenic mechanisms of TGF-β signaling. Previous studies have shown TGF-β signaling reduces proliferation, promotes apoptosis and differentiation and acts as a tumor suppressor in premalignant tumor development and as a tumor promoter in advanced tumors^65^. SMAD3 can form transcription complexes to regulate TGF-β target genes and loss of SMAD3 appears to promote colorectal tumorigenesis^66,67^. By utilizing RNA-seq data from HCT116 and DKO cell lines, we further verified the functional consequences of DNA methylation changes of selected gene for colorectal tumorigenesis (Fig. 5). Our results, although may not accurately reflect the role of blood DNA methylation alterations during tumorigenesis, suggest the different impacts of DNA methylation changes on the gene expressions of oncogenes (e.g. KRAS and SCL2A1) and tumor suppressor genes (e.g. ARHGEF4, EPHB2 and SOCS3). In order to fully elucidate the impact of DNA methylation alterations on CRC development and progression, further studies on the physiological function analysis of each genes is warranted.

Despite the above novel findings, some limitations in our study should be noted. Clearly, the sample size is relatively small. We took a more conservative approach to the data analysis. For example, we only selected 238 DMRs for functional analysis. Despite the encouraging initial CRC specific results, further work is warranted to validate these findings in a large cohort of patients.

Furthermore, there is a large difference between the mean ages of the CRC group and the obese or control groups. The association between age and the DNA methylation profile is previously reported. As indicated in the first paragraph of the Results section, age was considered as a covariate in the logistic regression model for detecting differential DNA methylation to control any distortion effect. Given the fact that the overlapping DMRs were identified separately from CRC and obesity and the obese group is age-matched to the control group, we can deduce that age has minimal effect on the overlapping DMRs. Further studies with age-matched CRC patients are needed to determine the relationship between age-related methylation changes and CRC susceptibility.

Finally, we acknowledge the heterogeneity of our sample as whole blood samples contain a mixture of various cells that exist in the blood circulation. Nonetheless, an interplay between cell types composing the whole blood exists and may have an implication for CRC development. It was thus important to assess whole blood rather than isolated plasma, serum and leukocytes including monocytes, macrophages and neutrophils. Moreover, the contribution of the cellular composition is accounted for by the total variation of DNA methylation measured. In clinical research, whole blood is one of the most readily available samples for biomarker analysis. Because under certain circumstances the amount of blood drawn from patients does not allow us to analyze the contribution of each component to DNA methylation changes in blood, a pooling method using DNA from groups of individuals has recently shown promise in identifying significant methylation markers^68^.

In summary, our study points to DNA methylation alterations linking obesity and CRC with the promise for early prognosis of CRC risk in relation to obesity. Our results provide additional information for deeper understanding of CRC development, and highlight potential new targets for prevention of CRC. Future research effort should include the integration of DNA methylation, gene expression and disease initiation and progression to provide comprehensive insight into the mechanisms through which obesity may drive cancer pathogenesis.

## Materials and Methods

### Study population

The study was approved by the Wright State University Institutional Review Board and all methods were performed in accordance with the relevant guidelines and regulations. Whole blood samples were obtained from either the Cooperative Human Tissue Network (CHTN) (15 CRC patients) and Advocate Sherman Hospital (5 CRC patients and 5 lean controls) (Table 1). The informed consent was collected by CHTN and Advocate Sherman Hospital. The DNA methylation data of whole blood samples from obese (n = 10) and lean controls (n = 10) was obtained from a publicly available database (NCBI GEO; accession number GSE85928). RNA-seq datasets for two replicates of HCT116 and DKO cells were obtained from GEO (accession number GSE60106).

### DNA extraction, RRBS library preparation and sequencing

Whole genomic DNA was extracted from whole blood using a DNeasy Blood & Tissue Kit (Qiagen, USA) following the manufacturer’s protocol. After checking the quality of the extracted DNA, 500 ng of genomic DNA was digested overnight with Msp1 (New England Biolabs, USA). The sticky ends produced by MspI digestion were filled with CG nucleotides, and 3′A overhangs were added. A DNA library was prepared using NEXTflex Bisulfite-Seq Kit (Bioo Scientific) following a standard procedure. A bisulfite conversion step was performed prior to PCR amplification using the EZ DNA Methylation-Gold kit (Zymo Research Corp.) following the manufacturer’s instructions. All PCR reactions for RRBS were purified using AMPure XP (Beckman Coulter, Brea, USA), and analyzed on a bioanalyzer. Sequencing was performed on the Illumina HiSeq.2500 for a paired-end 2 × 50bp run, with 150 million reads from each direction. Data quality check was done on the Illumina SAV. De-multiplexing was performed with the Illumina Bcl2fastq2 v2.17 program.

### Bioinformatics and statistical analysis

The quality of the raw reads was examined with FastQC. The adapter trimming and filtering of the high quality reads was carried out with Cutadapt v1.8.3 and Trim Galore v0.4.0 with the -RRBS option. Quality processed reads were mapped to human genome (hg19) using Bismark assisted by Bowtie2. Before DMC and DMR analyses, methylation calls were filtered by discarding bases with coverage below 5X and bases with more than 99.9th percentile coverage in each sample. CpG sites on sex chromosomes and mitochondrion were excluded from the analyses. Individual DMCs were identified between obesity/CRC and control groups using logistic regression with the R package methylKit. Read coverage was normalized between samples. A minimum of three individuals per group were required for a CpG site to be analyzed. The CpGs with at least 10% methylation difference and a q-value < 0.05 were considered to be differentially methylated. DMRs were determined using the R package eDMR with default parameters. To be considered significant, a DMR needed to contain at least one DMC, three CpG sites, and an absolute mean methylation difference greater than 5%. We annotated the DMRs identified using UCSC Refseq gene models with promoter regions defined as being 2 kb upstream from transcription start site (TSS). CpG islands were defined based on UCSC annotation (http://genome.ucsc.edu/). Functional Gene Ontology (GO) and Kyoto Encyclopedia of Genes and Genomes (KEGG) pathway enrichment analyses of involved genes were performed using DAVID bioinformatics resources (version 6.8; http://david.abcc.ncifcrf.gov/). The p-value was calculated using the modified Fishers exact test and the GO categories and KEGG pathways were identified as significantly enriched when p value was <0.05. Additional parameters were set to the default values. The Kruskal-Wallis test was used for comparison of DNA methylation levels among all groups, while the Mann-Whitney U test was used for comparison between the groups of subjects. A p value < 0.05 was defined as statistical significance and <0.1 was considered as marginal significance. Raw reads from RNA-seq were trimmed and mapped to human genome (hg19) using Tophat v2.1.1. Gene-level counts were generated using HTSeq v0.6.1 and also validated with Cufflinks v2.2.1. For differential expression analysis, read counts were normalized across libraries using the trimmed mean of M-values (TMM) method implemented in the R package edgeR v3.22.3 and were subsequently transformed to log2-counts per million (log-CPM) and corrected for heteroscedasticity with voom transformation of the R package limma v3.36.2. The log-CPM values of representative genes were visualized as heat map using the heatmap.2 function of the R package gplots v3.0.1 with ‘scale = row’ parameter.

## Data Availability

The datasets generated during and/or analyzed during the current study are not publicly available but are available from the corresponding author on reasonable request.
